# Identification and Validation of a Dysregulated miRNA-Associated mRNA Network in Temporal Lobe Epilepsy

**DOI:** 10.1155/2021/4118216

**Published:** 2021-10-22

**Authors:** Xing Li, Yunli Han, Dejun Li, Hai Yuan, Shiqin Huang, Xiaolan Chen, Yuanhan Qin

**Affiliations:** ^1^Departments of Pediatrics, The First Affiliated Hospital of Guangxi Medical University, Nanning, 530021 Guangxi Zhuang Autonomous Region, China; ^2^Departments of Pediatrics, Wuzhou Workers' Hospital, Wuzhou, 543000 Guangxi Zhuang Autonomous Region, China

## Abstract

**Objectives:**

This study is aimed at exploring the relationships between miRNAs and mRNAs and to characterize their biological functions in temporal lobe epilepsy (TLE).

**Methods:**

Novel clinical significant miRNAs and target genes and their potential underlying mechanisms have been discovered and explored by mining miRNAs and mRNA expression data of TLE patients using various bioinformatics methods. Reverse transcription-quantitative polymerase chain reaction (RT-qPCR) was used to validate the bioinformatic analysis results.

**Results:**

A total of 6 dysregulated miRNAs and 442 differentially expressed genes (DEGs) related to TLE were obtained from GEO database (GSE114701 and GSE127871 datasets). A protein-protein interaction (PPI) network containing the 442 DEGs was established. mRNA response elements from the 6 dysregulated miRNAs were predicted using the miRDB and TargetScan bioinformatic tools. By merging the identified targets of the dysregulated miRNAs and the 247 downregulated DEGs, a miRNA-mRNA network was constructed revealing the interaction of miR-484 with eight mRNAs (*ABLIM2*, *CEP170B*, *CTD-3193O13.9*, *EFNA5*, *GAP43*, *PRKCB*, *FXYD7*, and *NCAN*). A weighted correlation network analysis (WGCNA) based on the eight genes was established and demonstrated that these mRNAs, except *FXYD7* and *NCAN*, were hub genes in the network. Gene Oncology (GO) annotation and Kyoto Encyclopedia of Genes and Genomes (KEGG) pathway enrichment analysis revealed that the six hub genes were mainly involved in cellular-related biological functions and the neurotransmitter synapse pathway. The differences in expression levels of the miR-484 and the three hub genes (CTD-3193O13.9, EFNA5, and PRKCB) observed experimentally in TLE patients compared to those of healthy controls were consistent with the WGCNA prediction.

**Conclusion:**

Our study suggests that understanding the miRNA-mRNA interactions will provide insights into the epilepsy pathogenesis. In addition, our results indicate that miR-484 may be a promising novel biomarker for TLE.

## 1. Introduction

Temporal lobe epilepsy (TLE) is the refractory epilepsy marked by spontaneous recurrent seizures and the leading cause of hippocampal sclerosis. TLE is accountable for 50–80% of all diagnosed refractory epilepsy cases and is divided mainly into medial temporal lobe epilepsy (MTLE) and lateral temporal lobe epilepsy (LTLE) [1]. TLE neuropsychiatric complications have been reported to affect 30–70% of patients, the most common of which are memory deficits [2] and cognitive impairment [3]. Although neurostimulation, epileptogenic zone surgery, and antiepileptic drugs have been applied for TLE therapy, the drug-resistant rate and neuropsychological impairment of TLE patients are still high [4]. Recent studies have shown some new insights about genetic and regulatory changes in the biological processes underlying TLE. However, the precise pathogenesis and effective biomarkers involved in the TLE epileptogenesis still remain unclear. Thus, recognizing TLE-related molecules for early diagnosis and treatment is currently an urgent need.

MicroRNAs (miRNA) are a group of small noncoding RNAs (approximately 20 nucleotides) that modulate the posttranscriptional expression of the target gene. They specifically bind to target mRNAs in the untranslated region, which results in transcript degradation or translation inhibition. miRNAs have been continuously reported to participate in numerous biological processes in recent years, including several neurological diseases. In particular, different evidence has identified miRNA expression changes in epilepsy [5, 6]. For example, several studies have shown that miRNA can be manipulated to reduce spontaneous seizure and be a potential therapeutic target for TLE. A recent study reported a positive correlation between miRNA-145-5p and clinical evaluation of refractory epilepsy [7]. In addition, the miR-23a up-regulation can aggravate the hippocampal neuronal injuries and memory impairment in the mouse TLE model [8]. Therefore, the miRNA molecular functions on epileptogenesis and its progression have received considerable attention.

In this study, we analyzed miRNAs and mRNA expression profiles of TLE patients available in the GEO database and identified a set of differentially expressed genes (DEGs) and miRNAs. In addition, we collected miRNA-target interactions to construct a miRNA–mRNA network and acquired key TLE-related genes. By weighted correlation network analysis (WGCNA), we identified the key genes that are significantly associated with TLE clinical features. GO annotation and KEGG enrichment analysis were performed to unravel the biological mechanisms underlying TLE.

## 2. Materials and Methods

### 2.1. Microarray Data Collection

miRNA expression profiles of temporal lobe epilepsy (TLE) patients were collected from the NCBI GEO database (https://www.ncbi.nlm.nih.gov/geo/) using accession number GSE114701. In total, 40 samples (20 TLE patients after seizure and 20 healthy controls) analyzed on the TaqMan OpenArray Human microRNA Panel platform were included in each subseries datasets (GSE114697 Marburg and GSE114700 Beaumont). The miRNA expression file of normalized RT-qPCR data was downloaded and further processed. As for GSE114697 and GSE114700, microRNA expression in the plasma of 16 patients with epilepsy, before and after seizure, and 16 controls was measured by the TaqMan OpenArray Human microRNA Panel.

RNA-seq data of mRNA expression for hippocampal lesions in TLE were obtained from the GEO database using accession number GSE127871, which contains hippocampal tissue resected from 12 medically intractable TLE patients with presurgery seizure frequencies ranging from 0.33 to 120 seizures per month. Clinical data features, such as high seizure frequency (HSF) vs low seizure frequency (LSF) per month, were also collected on the same dataset. Data from 12 hippocampus samples that were analyzed on an Illumina HiSeq 2500 platform were also included. These samples contain 4 HSF patients (mean = 4 seizures/month) and 8 LSF patients (mean = 60 seizures/month).

### 2.2. Measures

#### 2.2.1. Identification of Differentially Expressed Genes (DEGs) and miRNAs

Human miRNAs were obtained from GSE114697 and GSE114700 datasets, and differentially expressed miRNAs were identified using the 2^−ΔΔ*Ct*^ method. The significant differences between groups (HSF and LSF patients) were analyzed using the paired *t*-test. The miRNAs that met both criteria of adjusted *P* < 0.05 and ∣log2(FC) | >1 were considered as TLE-related differentially expressed miRNAs. All differentially expressed miRNAs were integrated in the Marburg and Beaumont centers.

Differentially expressed genes (DEGs) in the GSE127871 dataset were identified using edge R, a Bioconductor package that employs the empirical analysis method. Significant DEGs between the HSF and LSF groups met both criteria of a *P* value < 0.05 and ∣log2(FC) | >1.5.

### 2.3. Functional and Pathway Enrichment Analysis

The DEGs identified in the GSE127871 dataset were classified by the related terms of gene ontology (GO) analysis, including cellular components (CC), molecular function (MF), and biological processes (BP). Kyoto Encyclopedia of Genes and Genomes (KEGG) is considered an advanced database that integrates genomic information, biological functions, disease development, and several bioinformatics studies. Based on the GO and KEGG databases, Fisher's exact test was used to identify relevance of DEGs and their functional items, especially the BP terms and functionally enriched KEGG pathways. A *P* value < 0.05 was considered a statistically significant enrichment.

### 2.4. Establishment of the Protein-Protein Interaction (PPI) Network and Identification of Hub Genes

The Search Tool for the Retrieval of Interacting Genes (STRING) database (https://string-db.org/) was used to analyze protein-protein interaction (PPI) information [9]. The PPI pairs were extracted using an interaction score > 0.9 and were applied for the PPI network construction. Subsequently, the Cytoscape software (version 3.6.1; http://cytoscape.org/) was used to visualize the PPI network [10]. Notes with a higher degree of connectivity represented the highly interacting genes or proteins in the network. CytoHubba is a common useful plugin for calculating the degree of each node in Cytoscape. In this study, nodes with a degree greater than 5 were identified as central genes in the network.

### 2.5. Construction of the miRNA–mRNA Network

The miRNA-mRNA interactions were predicted by miRDB and TargetScan, two algorithms for miRNA target prediction. After obtaining miRNA-mRNA regulatory data, the network of association between miRNA and mRNA networks in TLE was generated through the intersection of the predicted miRNA target genes and the identified DEGs. The Cytoscape software was employed to visualize the regulatory network.

### 2.6. Screening of Related Modules to Seizure Risk by Weighted Correlation Network Analysis (WGCNA)

WGCNA is a data mining approach used to screen biomarkers and pathways implicated in the pathogenesis, progression, and prognosis of various diseases. WGCNA can be used for finding clusters (modules) of highly correlated genes, for summarizing such clusters using the module eigengene or an intramodular hub gene, for relating modules to one another and to external sample traits (using eigengene network methodology), and for calculating module membership measures [11, 12]. The WGCNA algorithm was used to analyze the correlation between the modules and clinical parameters in TLE. The coexpression network was constructed using the WGCNA R package and visualized by the Cytoscape software. Then, seizure risk-related modules were identified using the WGCNA algorithm. Briefly, Person's correlation coefficients were performed to calculate the counts per million (CPM) in selected genes. The CPM matrix was used as the input of the expression value for the hierarchical clustering analysis. Subsequently, the gene modules that were significantly associated with clinical traits of seizure frequency were identified. The gene information in the most relevant module was extracted, and the potential biological terms and pathways were investigated by GO annotation and KEGG pathway enrichment analysis.

### 2.7. miRNA-484 and Hub Gene Validation

Plasma samples were obtained from 20 patients diagnosed with TLE and 20 healthy controls at the First Affiliated Hospital of Guangxi Medical University. Total RNA was isolated from samples using Trio (Invitrogen) according to the manufacturers' protocol. The total RNA was measured using a Nanodrop 2000 microvolume spectrophotometer (LifeReal, China) by absorbance measurements at a 260 nm wavelength. RNA integrity was analyzed by 2% agarose gel electrophoresis stained with ethidium bromide. Complementary DNA (cDNA) was synthesized from 1 *μ*g of total RNA using the TransScript® Uni One-Step gDNA Removal and cDNA Synthesis SuperMix (TransGen Biotech, China) according to the manufacturer's guidelines. The reverse transcription-PCR (RT-PCR) reactions were carried out on an Agilent AriaMx Real-Time PCR system with a 20 *μ*L reaction volume. The PCR primer sequences of miRNA-484 and its target genes are shown in Table 1. To create the RT-PCR normalization, U6 RNA and GAPDH were chosen as the endogenous control for miRNA and mRNA, respectively. miRNA-484 and six DEG expression were determined using the 2^−ΔΔ*Ct*^ method. All RT-PCR procedures were performed in triplicates. The results were analyzed by Mx3000P real-time PCR software version 2.00.

## 3. Results

### 3.1. Identification of Differentially Expressed Genes (DEGs) and miRNAs

**Figure 1:**
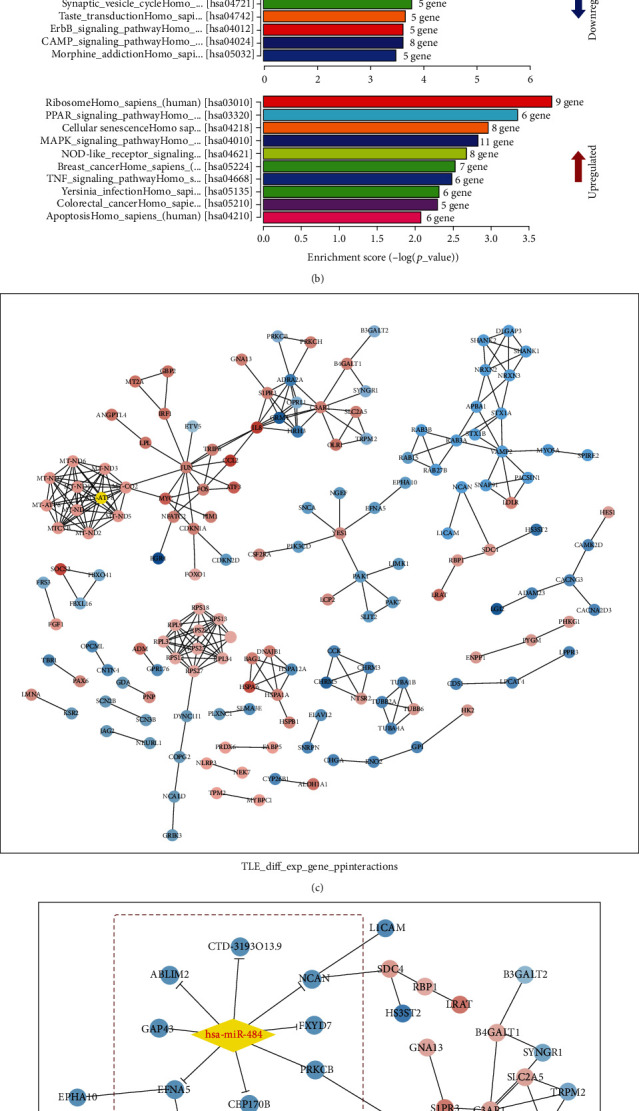
(a) The volcano plot of DGEs in TLE based on data from GEO. (b) Biological pathway enrichment analysis of up- and downregulated DEGs with the top 10 enrichment scores. (c) Protein–protein interaction network constructed with the DEGs. Red nodes represent upregulated genes, and blue nodes represent downregulated genes. (d) An integration network of the miRNA-mRNA regulatory network and PPI network. The network consisting of has-miRNA-484, 76 upregulated DEGs, and 80 downregulated DEGs was generated by Cytoscape. The yellow diamond represents has-miRNA-484, red nodes represent upregulated genes, blue nodes represent downregulated genes, and the T-arrow edge represents miRNA-mRNA interactions.

A total of 442 DEGs were identified by comparing HSF and LSF hippocampus tissues from the GSE127871 dataset, with 195 and 247 DEGs being up- and downregulated, respectively. As shown in Figure 1(a), the expression of the identified DEGs potentially distinguishes the HSF group from the LSF group by a volcano plot. The volcano plot was generated by the R package “ggplot2.” The red and green dots represent the up- and downregulated DEGs, respectively. In addition, 51 dysregulated miRNAs were identified in the GSE114697 and GSE114700 datasets, among which 6 (has-miR-133a, has-miR-17, has-miR-191, has-miR-223, has-miR-328, and has-miR-484; Table 2) showed to have a significant differential expression, being upregulated in TLE patients (∣log2(FC) | >1, *P* value < 0.05).

### 3.2. Functional Enrichment Analysis of DEGs

GO and KEGG analyzes were performed at the DAVID server to understand the function of DEGs. According to the results of the GO term analysis, the most enriched BP terms of the upregulated DEGs were mainly related to the response to external stimulus, response to organic substance, and regulation of the developmental process. However, nervous system development, chemical synaptic transmission, and regulation of membrane potential were the most enriched items of the downregulated DEGs. KEGG analysis results revealed 35 pathways related to the upregulated DEGs and 64 associated with the downregulated DEGs. The upregulated DEGs were mainly involved in ribosome biogenesis, PPAR signaling, and cellular senescence pathways. The downregulated DEGs, in turn, were mainly associated with axon guidance, insulin secretion, and neuroactive ligand-receptor interaction pathways.  Figure 1(b) shows the top 10 most enriched pathways for up- and downregulated DEGs.

### 3.3. PPI Network Analysis of DEGs

Based on the STRING database, a PPI network was constructed containing all 442 identified DEGs and that included 268 interactions according to the highest confidence score and using a combined score > 9 as the cutoff. After calculating the connectivity degree, the PPI network was built comprising 156 nodes and 268 edges and the interactions between the DEGs were visualized using the Cytoscape software (Figure 1(c)). Based on the CytoHubba plugin calculation, DEGs with a degree > 6 were considered central genes in the PPI network. The top 28 central genes are shown in Table 3. Among them, *VAMP2* and *ADRA2A* were downregulated genes, while others were all upregulated in TLE samples.

### 3.4. Identification of Eight miRNA–mRNA Interactions

The miRNA–mRNA interactions were predicted with the miRDB and TargetScan algorithms. Following the collection of overlapping genes between the target genes of the six upregulated miRNAs and the 247 downregulated DEGs, the miRNA-mRNA network of TLE was constructed. miR-484 was found to have the highest overlapping target number with DEGs. The intersection between the miRNA-mRNA network and the DEG PPI network provided a preliminary view of the connections between miR-484 and DEGs. Moreover, eight miRNA–mRNA regulatory modules, including the miR-484/FXYD7regulatory axis, miR-484/NCAN regulatory axis, miR-484/ABLIM2 regulatory axis, miR-484/CEP170B regulatory axis, miR-484/CTD-3193O13.9 regulatory axis, miR-484/EFNA5 regulatory axis, miR-484/GAP43 regulatory axis, and miR-484/PRKCB regulatory axis, were found in the intersected network (Figure 1(d)).

### 3.5. Coexpression Module Identification Based on WGCNA Analysis

**Figure 2:**
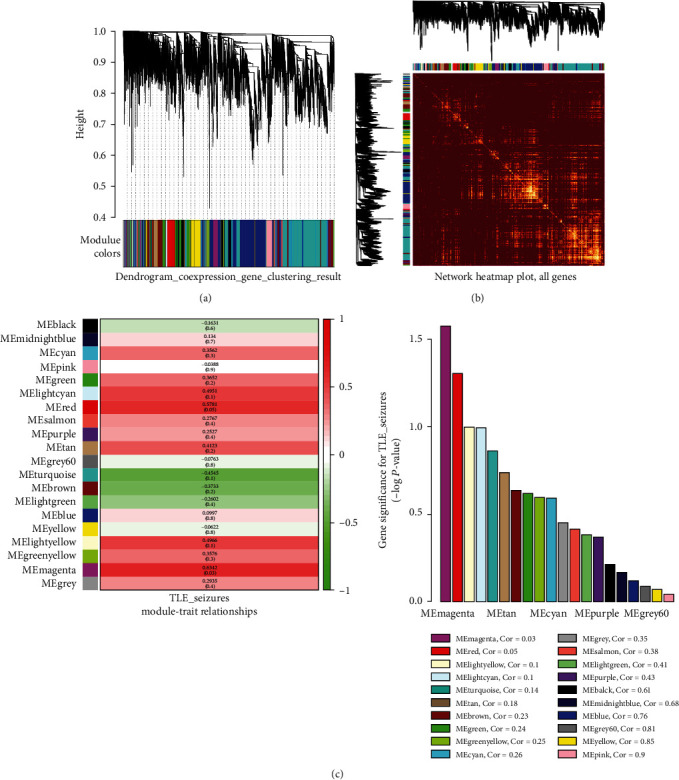
Construction of coexpression modules by WGCNA package in R. (a) The cluster dendrogram of coexpression genes in GSE127871. Each color row represents one coexpression module and gray represents genes that cannot be categorized into any module. (b) Heat map plot of genes in the weighted correlation network. Each colored row represents a color-coded module which contains a group of highly connected genes. (c) Heat map of the module-trait relationship. The module name is shown on the left side of each cell. The intensity of correlations is indicated on the right side of the heat map. Red represents the negative correlation between modules and the seizure frequency of TLE, and green represents negative correlation.

The GSE127871 dataset containing 12 TLE samples was used for hierarchical clustering with the R WGCNA package. As shown in Figure 2(a), a total of 20 coexpression modules with a comfortable scale-free topology fit index (value = 0.8) were identified in the hierarchical clustering dendrogram. The gene counts varied for each module from 48 to 3127 genes.

To analyze the interaction association between the different modules, a heat map was generated using the “heat map tool” package in R.  Figure 2(b) revealed that a light color density in the middle of the figure represents a high correlation of different modules. In addition, the module–trait relationship analysis results showed that the turquoise and magenta modules were strongly related to TLE seizures, as suggested in Figure 2(c).

To screen for genes associated with the upregulated miRNA-484 in TLE, the turquoise module was selected for subsequent analysis. It was found that six genes (*ABLIM2*, *CEP170B*, *CTD-3193O13.9*, *EFNA5*, *GAP43*, and *PRKCB*) were enriched in the turquoise module according to their degree of connectivity (*R*^2^ = 0.44). Thus, these six genes were recognized as the most significant genes related to the TLE illness status and then selected as hub genes.

### 3.6. GO Annotation and KEGG Pathway Analyzes of Hub Genes

**Figure 3:**
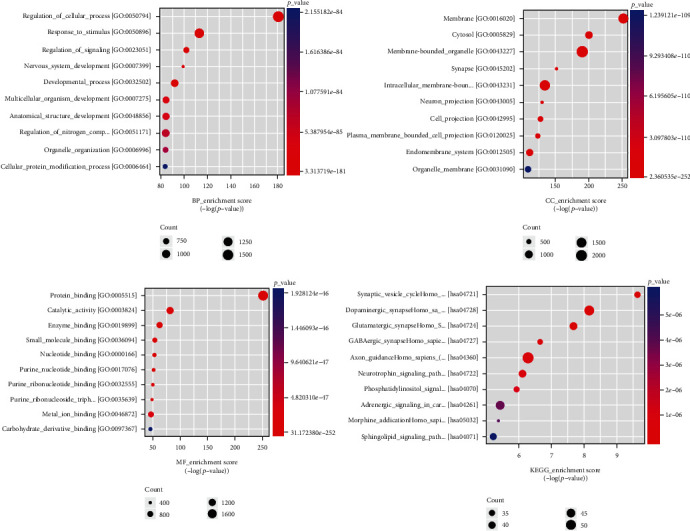
Top ten GO and KEGG enrichment annotations of the turquoise module. BP, CC, and MF terms and KEGG pathway enrichment analysis. The sizes of the circle dots represent the counts of enriched DEGs, and the intensity of circle color represents the *P* value.

To further elucidate the TLE biological mechanism and identify potential biomarkers, GO annotation and KEGG pathway analyzes of the six hub genes were performed. The enrichment and *P* value analyses revealed the top ten highly enriched BP, CC, and MF terms, which are shown in Figure 3. It was observed that the most significantly modulated GO terms in BP were cellular process (*P* = 3.31*e* − 181), response to stimulus (*P* = 1.34*e* − 113), and regulation of signaling (*P* = 1.74*e* − 102). The CC enriched in TLE were primarily associated with the membrane (*P* = 2.36*e* − 252), cytosol (*P* = 4.56*e* − 201), and membrane-bounded organelle (9.30*e* − 191), while the enriched MF were primarily those associated with protein binding (*P* = 3.17*e* − 252), catalytic activity (*P* = 3.45*e* − 82), and enzyme binding (*P* = 4.52*e* − 61). In addition, these hub genes also participate in the regulation of many KEGG pathways. A total of 110 significantly enriched pathways were obtained (*P* value < 0.05). The top 10 *P* values of enriched pathways are also shown in Figure 3. Among them, the synaptic vesicle (SV) cycle (*P* = 2.32*e* − 8210), dopaminergic synapse (*P* = 6.97*e* − 8209), and glutamatergic synapse (*P* = 2.10*e* − 8208) were most strongly associated pathways with TLE.

### 3.7. Experimental Validation of miRNA-484 and Hub Genes

**Figure 4:**
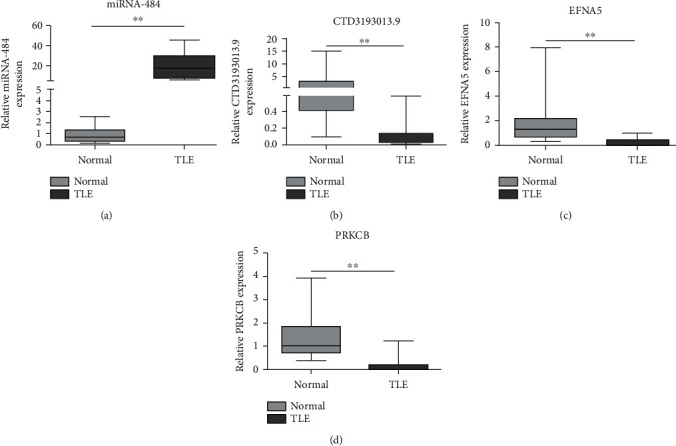
Expression levels of miRNA-484 and six genes in TLE: (a) miRNA-484, (b) *CTD-3193O13.9*, (c) *EFNA5*, and (d) *PRKCB*. The mark ^∗∗^ means that there was significant difference between TLE and controls.

To confirm the results obtained by the bioinformatics tools, the expression levels of the miRNA-484 and the six hub genes were verified in the plasma of 20 TLE patients and compared to those of 20 healthy controls by RT-qPCR. The results showed that the miRNA-484 expression level was significantly increased in TLE patients compared to controls (Figure 4(a)). Moreover, the expression level of the *CTD-3193O13.9*, *EFNA5*, and *PRKCB* genes decreased significantly in TLE patients (Figures 4(b)–4(d))). The differences in the expression level of the *ABLIM2*, *GAP43*, and *CEP170B* genes between the two groups were not significant. These results revealed that the increased levels of miRNA-484 expression and decreased levels of hub gene expression (*CTD-3193O13.9*, *EFNA5*, and *PRKCB*) were consistent with the data obtained by the WGCNA analysis.

## 4. Discussion

TLE is a heterogeneous disease and a unique subtype of refractory epilepsy with poor prognosis. TLE patients are more likely to have drug resistance and underwent cognitive impairment. As the molecular mechanisms underlying therapy resistance and pathological process of TLE have not been fully elucidated, there is still no specific therapeutic target and an effective prognostic factor. Therefore, more studies are needed to discover and explore new TLE-related molecules.

MicroRNAs are a group of small noncoding RNAs with abundant biological information that have been shown to be involved in a myriad of human disorders, including seizure disorders. Early functional studies on TLE indicated that miRNA usually serves as diagnostic and therapeutic targets to influence seizures or hippocampal pathology. For example, miRNA-124, which is related to neuronal differentiation, can inhibit neuronal excitability by targeting cAMP-response element-binding protein1 (CREB1) [13]. Moreover, miR-199a-5p silencing inhibits the hippocampal neuron loss and apoptosis by targeting the antiapoptotic protein silent information regulator 1 (SIRT1) [14]. Emerging studies on the multitargeting effects of miRNAs and the coordinating gene networks have also revealed that miRNAs are crucial TLE regulators. However, the mechanism of miRNA in TLE remains unclear. There is still a need for future identification of new dysregulated miRNAs and miRNA targets in TLE.

In the present study, we identified a group of differentially expressed molecules correlated to TLE in the publicly available databases using different bioinformatics tools. In the identified molecules, 6 miRNAs (has-miR-133a, has-miR-17, has-miR-191, has-miR-223, has-miR-328, and has-miR-484) were upregulated, 195 DEGs were upregulated, and 247 were downregulated. After the construction of a miRNA–mRNA regulatory network, we found that the upregulated miR-484 is able to regulate the expression of eight downregulated DEGs, including *ABLIM2*, *CEP170B*, *CTD-3193O13.9*, *EFNA5*, *GAP43*, *PRKCB*, *FXYD7*, and *NCAN*. These data suggest that miR-484 may be a potential targeted marker for TLE. This is the first report in the literature indicating that miR-484 plays a role in epilepsy.

To ascertain whether hsa-miR-484 performs functions and influences the clinical phenotypic effects in TLE, we carried out a WGCNA analysis and found that the eight identified DEGs enriched the turquoise, brown, and black modules associated with TLE. Among them, the turquoise module was the most strongly related to the TLE seizure phenotype according to the module–trait relationship analysis. Remarkably, we found that, except *FXYD7* and *NCAN*, all six other downregulated DEGs that are modulated by miR-484 were mostly enriched in the significant turquoise module and can function as hub markers for TLE. Finally, GO functional annotations and KEGG pathway analysis of these six hub genes in the turquoise module were performed to understand the mechanisms underlying epileptogenesis. The results showed that these hub genes were involved in multiple GO BP terms such as the cellular process, multicellular organism development, and cellular protein modification process. In addition, these hub genes also participate in the regulation of many KEGG pathways, such as the SV cycle, dopaminergic synapse, GABAergic synapse, axon guidance, glutamatergic synapse, neurotrophic signaling, phosphatidylinositol signaling system, and sphingolipid signaling. As an example, animal model experiments reported that the SV cycle was targeted with some pathogenic genes, which then derived neurotransmitters release—monoamines, glutamate, GABA, etc.—and participated in the pathophysiology of seizures and epilepsy [15, 16]. Evidence has indicated that dopamine can aggravate epileptiform activity by decreasing the Mg^2+^ concentration in the newborn mouse hippocampus in vitro. Furthermore, the induction of changes in the GABAergic and glutamatergic systems leads to increased neuron excitability and the involvement of these dysregulated systems in epileptogenesis [17]. Glutamate receptors, including AMPA receptors, are characterized by influencing the alternation of Ca^2+^ concentrations, thus causing neuronal death and becoming involved in the epilepsy pathophysiology [18, 19]. Experiments using the KA-induced TLE mouse model have found that a reduction in AMPA receptors leads to a decrease in glutamatergic transmission and therefore increases the neuron excitotoxicity and compromises the hippocampal cognitive functions [19]. These findings provide important evidence for the regulatory mechanism of miRNA-484 and the six hub genes (*ABLIM2*, *CEP170B*, *CTD-3193O13.9*, *EFNA5*, *GAP43*, and *PRKCB*) in TLE.

Previous studies have shown that three of the six hub genes identified here play key roles in epilepsy. Shu et al. suggested that ephrin-A5 (*EFNA5*) is upregulated in hippocampus tissue of a TLE mouse model, and that modulates neuron generation and microvessel remodeling by inhibiting the ERK and Akt signaling pathways [20]. Ying et al. found that *GAP43* expression is higher in the epileptic area compared to the nonepileptic area. They also showed that patients with higher *GAP43* scores are associated with longer epilepsy duration and poor surgical outcome in focal cortical dysplasia IIA/B [21]. Danis et al. reported that the *PRKCB* expression is reduced by miR-184 in the 3′UTR luciferase reporter assay and that this effect may be involved in the TLE translational regulation [22]. Although the involvement of the *ABLIM2* gene in epilepsy has never been previously reported, it has been associated with neuron guidance processes [23]. Furthermore, bioinformatics analyzes have shown that *ABLIM2* is a hub gene and plays an important role in neurodegenerative diseases [24]. However, in relation to the other two identified hub genes (CEP170B and CTD-3193O13.9), their involvement in epilepsy and other neurological disorders had not been pointed out so far.

RT-qPCR analysis of the miRNA-484 and the six hub gene expression levels in the plasma of TLE patients confirmed the results obtained by bioinformatic analyzes. The data revealed that the miRNA-484 expression was significantly increased in TLE patients. The results also revealed that three of the six hub genes (*CTD-3193O13.9*, *EFNA5*, and *PRKCB*) were associated with TLE and had a significant decrease in their expression level compared to the controls. These results indicate that miRNA-484 can inhibit seizure frequency in TLE by targeting *CTD-3193O13.9*, *EFNA5*, and *PRKCB*, which suggests that miRNA-484 may be a promising diagnostic marker for epileptogenesis. Although the experimental verification of the expression levels of miR-484 and the three hub genes in TLE patients corroborates the bioinformatics data and indicates their involvement in the TLE pathogenesis, further studies should be performed to confirm our findings in vitro and in vivo.

## 5. Conclusions

We identified six hub genes, the majority of which were associated with the seizure development and enrichment of the synaptic structure and neurotransmitter signaling pathways. In addition, we found that miR-484 upregulation is accompanied by the epilepsy progression via inhibition of the three hub gene expression (*CTD-3193O13.9*, *EFNA5*, and *PRKCB*). Our results highlight the possibility that miR-484 may be indicated as a promising novel biomarker for TLE.

## Figures and Tables

**Table 1:** Primer sequences of PCR.

Gene ID	Forward primer (5′-3′)	Reverse primer (3′-5′)
hsa_miRNA_484	TCAGGCTCAGTCCCCTCC	CAGTGCGTGTCGTGGAGT
ABLIM2	GGGAGGATGGAAGCTTGGAC	GTCCTGGGAGAGGGTCAGAT
CEP170B	AAGATGAGTGCCACGTCCTG	CAGCGTGACGTACTTCTGGT
CTD-3193O13.9	CCGAGAGGAACTACAGCGTC	CACCTCCATCGCGGACAG
EFNA5	GCACGCTTCTCTCCATCTTGTG	AATGAAAGTGGGCGAGAAAGGA
GAP43	GCTGTGCTGTATGAGAAGAACC	AGGACTTTGTCATCGCCAGT
PRKCB	CGATTTTTCACCCGCCATCC	CACCACAATAGCCGTTGAGC

**Table 2:** Information of the six differentially expressed miRNAs in TLE.

miRNA	log2FC	*P* value	*q* value	Regulation
hsa-miR-133a	1.00434	0.0428	0.9061	Up
hsa-miR-17	1.33481	0.0063	0.0644	Up
hsa-miR-191	1.53602	0.0091	0.0666	Up
hsa-miR-223	1.08246	0.0057	0.0644	Up
hsa-miR-328	1.64252	0.0018	0.0435	Up
hsa-miR-484	1.66121	0.0016	0.0435	Up

**Table 3:** The top 28 differentially expressed genes in TLE.

Gene	Degree	Gene	Degree	Gene	Degree	Gene	Degree
JUN	12	MT-ND6	10	RPS18	9	RPL37	8
MT-CO2	11	MT-ND2	10	RPS13	9	VAMP2	8
C3AR1	11	MT-ND3	10	RPS29	9	IL8	8
MT-ND1	10	MT-ND5	10	RPS12	9	ADRA2A	8
MT-CYB	10	MT-ND4L	10	RPL9	9	TPT1	7
MT-ND4	10	RPS27	10	MT-ATP8	9	S1PR3	7
MT-ATP6	10	RPS23	9	RPL34	8	FOS	6

## Data Availability

The datasets generated and analyzed during the current study are publicly available in the GEO database (under accession code: GSE114701 and GSE127871). All the other raw data could be accessed by contacting the corresponding author if any qualified researcher needs it.
